# P-2203. Cytomegalovirus Reactivation in Patients Treated with Bispecific Antibodies (BsAbs)

**DOI:** 10.1093/ofid/ofaf695.2366

**Published:** 2026-01-11

**Authors:** Tori Pravato, Nina Orsini, Jungwook Kang, Krishna Shah, Yuxuan Li, Susan K Seo, Genovefa Papanicolaou

**Affiliations:** RWJ Trinitas Regional Medical Center, Staten Island, NY; Memorial Sloan Kettering Cancer Center, New York, New York; Memorial Sloan Kettering Cancer Center, New York, New York; Memorial Sloan Kettering Cancer Center, New York, NY, USA, NYC, New York; Memorial Sloan Kettering Cancer Center, New York, New York; Memorial Sloan Kettering, New York, NY; Memorial Sloan Kettering Cancer Center, New York, New York

## Abstract

**Background:**

Use of BsAbs for acute lymphocytic leukemia and lymphoma has been associated with cytomegalovirus (CMV) viremia and end-organ disease (EOD), with increased risk due to patients' underlying malignancies, prior chemotherapies, treatment-related complications (e.g., cytokine release syndrome), and prolonged cytopenias. There is no standard for CMV monitoring or prophylaxis after BsAb therapy. The primary objective of the study was to estimate the rate of CMV viremia following treatment with BsAbs.

**Figure:**
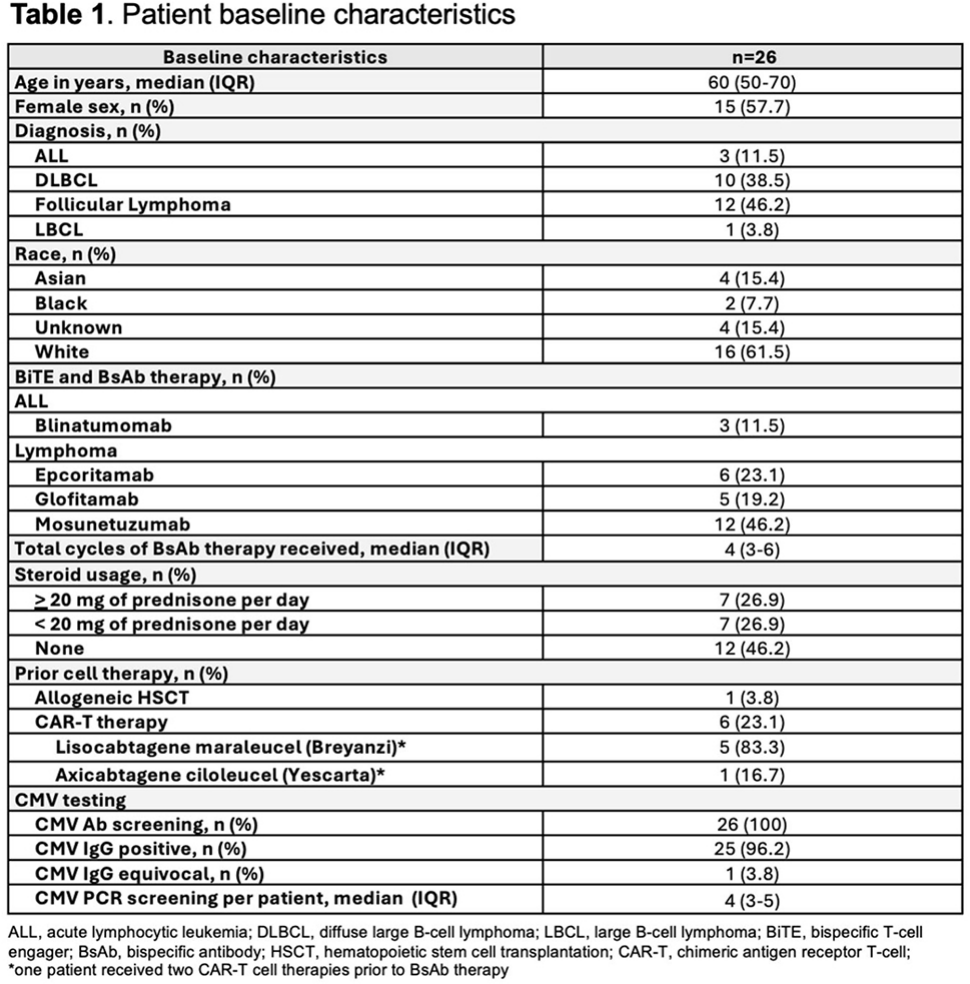

**Figure:**
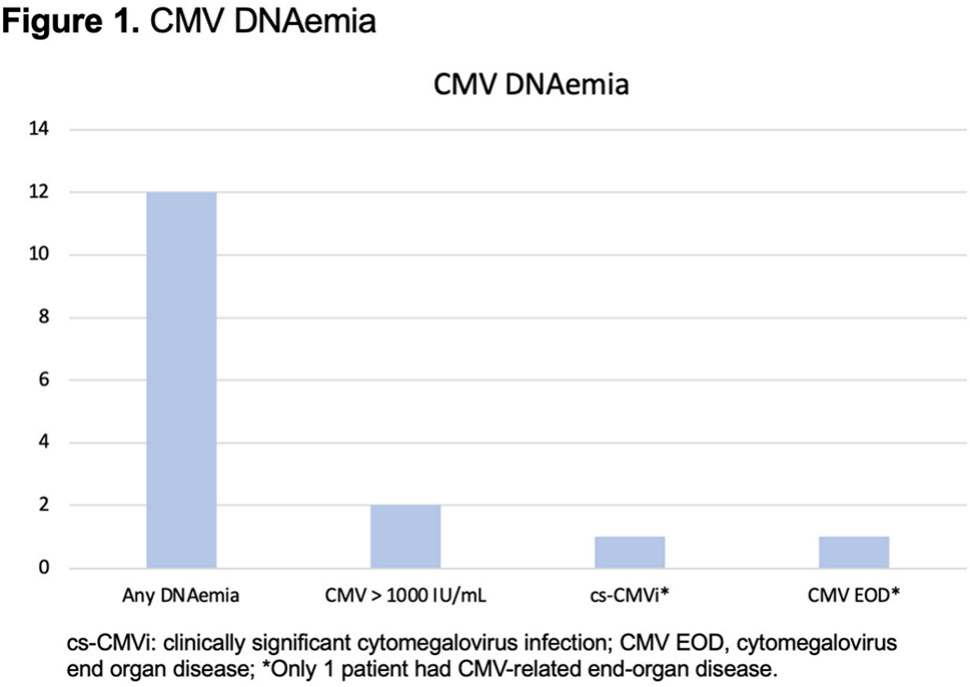

**Methods:**

A total of 68 patients treated with blinatumomab, epcoritamab, glofitamab, or mosunetuzumab for leukemia and lymphoma were screened for CMV IgG from 09/2024 to 04/2025. Twenty-six CMV-seropositive adults were included and monitored prospectively for CMV by a quantitative PCR in the plasma (lower limit of detection 34.5 IU/mL) at least monthly from first BsAb infusion through 6 months, receipt of allogeneic hematopoietic stem cell transplantation (HSCT) or chimeric antigen receptor T-cell (CAR-T) therapy, or death, whichever occurred first. Demographics and BsAb type and doses were extracted from patient records. The dose and duration of concomitant steroids and antiviral treatment were collected. CMV viral loads, CMV-directed treatment, and outcomes were captured.

**Results:**

Of 26 patients analyzed, the median follow-up after first BsAb infusion was 3 months (IQR, 2-4). Seven (27%) patients received steroids at doses > 20 mg of prednisone equivalent per day, and one (4%) patient had a history of prior allogeneic HSCT. Ten (38%) patients developed detectable CMV viremia at a median 18.5 days (IQR, 15-22) from first BsAb infusion. Subsequent PCR tests indicated clearance or stable detection < 1000 IU/mL. Two (8%) patients who received high dose steroids with no previous HSCT had maximum CMV PCR levels above 1000 IU/mL, and only one (4%) required treatment with (val)ganciclovir for CMV colitis for a total of 26 days. There was no CMV-related mortality.

**Conclusion:**

One-third of patients had transient low level CMV viremia that resolved spontaneously. One (4%) patient had CMV-related EOD. Despite increasing BsAb use, low rates of CMV reactivation were observed. Further research is ongoing.

**Disclosures:**

Genovefa Papanicolaou, MD, AICuris: Honoraria|Merck & Co Inc.: Grant/Research Support|Merck & Co Inc.: Consulting & other fees|Pulmoncide: Consulting and other fees|Scynexis: Consulting & other fees|Symbio: Consulting and other fees

